# EV20/NMS-P945, a Novel Thienoindole Based Antibody-Drug Conjugate Targeting HER-3 for Solid Tumors

**DOI:** 10.3390/pharmaceutics13040483

**Published:** 2021-04-02

**Authors:** Emily Capone, Rossano Lattanzio, Fabio Gasparri, Paolo Orsini, Cosmo Rossi, Valentina Iacobelli, Vincenzo De Laurenzi, Pier Giorgio Natali, Barbara Valsasina, Stefano Iacobelli, Gianluca Sala

**Affiliations:** 1 Department of Innovative Technologies in Medicine & Dentistry, University of Chieti-Pescara, 66100 Chieti, Italy; emily.capone@unich.it (E.C.); rossano.lattanzio@unich.it (R.L.); delaurenzi@unich.it (V.D.L.); 2 Center for Advanced Studies and Technology (CAST), Via Polacchi 11, 66100 Chieti, Italy; cosmo.rossi@unich.it; 3 Nerviano Medical Sciences Srl, 20014 Milan, Italy; Fabio.Gasparri@nervianoms.com (F.G.); Paolo.Orsini@nervianoms.com (P.O.); Barbara.Valsasina@nervianoms.com (B.V.); 4 Department of Gynecology and Obstetrics, Catholic University, 00168 Rome, Italy; vale.iacobelli@gmail.com; 5 MediaPharma s.r.l., Via della Colonnetta 50/A, 66100 Chieti, Italy; natalipg2002@yahoo.it

**Keywords:** antibody–drug conjugates, HER-3, targeted therapy, duocarmycin

## Abstract

HER-3 is becoming an attractive target for antibody–drug conjugate (ADC)-based therapy. Indeed, this receptor and its ligands are found to be overexpressed in several malignancies, and re-activation of its downstream signaling axis is known to play a critical role in modulating the sensitivity of targeted therapeutics in different tumors. In this study, we generated a novel ADC named EV20/NMS-P945 by coupling the anti-HER-3 antibody EV20 with a duocarmycin-like derivative, the thienoindole (TEI) NMS-P528, a DNA minor groove alkylating agent through a peptidic cleavable linker. This ADC showed target-dependent cytotoxic activity in vitro on several tumor cell lines and therapeutic activity in mouse xenograft tumor models, including those originating from pancreatic, prostatic, head and neck, gastric and ovarian cancer cells and melanoma. Pharmacokinetics and toxicological studies in monkeys demonstrated that this ADC possesses a favorable terminal half-life and stability and it is well tolerated. These data support further EV20/NMS-P945 clinical development as a therapeutic agent against HER-3-expressing malignancies.

## 1. Introduction

In recent years, antibody–drug conjugates (ADCs) have become promising antitumor agents in the context of personalized medicine [1,2]. These therapeutics are developed through the conjugation of antibodies to highly potent cytotoxic drugs. To date, nine ADCs have been approved for clinical use, and almost eighty different ADC candidates are being tested in phase I to III clinical trials [3]. Many ADCs target growth factor receptors expressed at the surface of cancer cells [4].

HER-3 belongs to the epidermal growth factor receptor family of tyrosine kinases (ErbB). The family consists of four members: HER-1 (ErbB-1), HER-2 (ErbB-2), HER-3 (ErbB-3) and HER-4 (ErbB-4). Many studies have demonstrated a critical role for ErbB receptors in cell survival, proliferation and differentiation, as well as malignant transformation [5,6], and therapeutic agents against HER-1 and HER-2 are considered among the most successful examples of targeted therapy. Indeed, both small molecules inhibiting the tyrosine kinase activity and therapeutic monoclonal antibodies targeting EGFR have been approved for clinical use in NSCLC (Non Small Cell Lung Cancer) and colorectal cancer, whereas agents targeting HER-2 are approved for HER-2-overexpressing breast and gastric cancer. Among these, two different anti-HER-2 antibody–drug conjugates (ADCs) are now approved, Kadcyla and Enhertu, with additional HER-2-targeted ADCs currently in phase III clinical trials. Moreover, recent studies have highlighted a key role for HER-3 in tumor progression and drug resistance [7]. Elevated expression of HER-3 has been observed in a wide variety of human cancers and has been associated with a worse survival in cancer patients with solid tumors. Studies on the underlying mechanisms implicate HER-3 expression as a major cause of treatment failure in cancer therapy. Activation of HER-3 signaling has also been shown to promote cancer metastasis [8]. These data strongly support the notion that therapeutic inactivation of HER-3 and/or its downstream signaling is required to overcome treatment resistance and improve the outcomes of cancer patients. Unlike the other family members, the functions of HER-3 are not mediated by enzymatic catalytic activity, indicating that the ATP analog class of TKIs would not be suitable for this target. The only possibility of blocking HER-3 is by targeting its extracellular domain (ECD) by specific antibodies [9].

Previously, we developed a HER-3 targeting ADC composed of EV20, a humanized anti-HER-3 antibody [10,11,12,13], conjugated with the tubulin inhibitor monomethyl auristatin F (MMAF) via a non-cleavable linker. This ADC, named EV20/MMAF, showed potent therapeutic activity against melanoma and breast cancer, in particular on a variant with acquired resistance to anti-HER2 therapies [14,15]. In the present paper, we have generated a novel EV20-based ADC, named EV20/NMS-P945, consisting of EV20 conjugated with a duocarmycin-like thienoindole derivative NMS-P528, using a peptide cleavable linker. There is a growing body of evidence suggesting that DNA-damaging agents, such as duocarmycins, have potential advantages over the microtubule-targeting agent MMAF as a loading agent for ADC development; first, DNA-damaging agents show higher cell killing activity, enabling ADCs to efficiently target tumor cells expressing low amounts of the antigen; secondly, these cytotoxic agents are active throughout the various cell-cycle phases, demonstrating high potency against both dividing and nondividing cells [16]. Moreover, in contrast to MMAF, which is hydrophilic and cannot permeate cell membranes, the NMS-P945 payload possesses bystander activity, which enables an efficient targeting of heterogeneous solid tumors [17]. Duocarmycins have been explored for ADC production in the past due to their high cytotoxic activity; however, their potential as payloads for ADC production has been hampered by unfavorable physicochemical properties, such as low solubility, causing poor conjugation levels and aggregation of the final products. More recently, ADC SYD985, a duocarmycin derivative conjugated to trastuzumab reached phase III clinical trials in HER2-positive breast cancers [18,19], confirming the validity of this class of toxins as ADC payloads. NMS-P528 is a new duocarmycin-like molecule developed at Nerviano Medical Sciences (NMS), which is suitable for ADC production due to its favorable physicochemical properties [17]. NMS-P945, a drug linker containing NMS-P528, proved to be suitable for antibody conjugation, obtaining monomeric ADCs with an average Drug-to–Antibody Ratio (DAR) > 3.5 [17]. In the present study, we explored the anti-cancer effects of EV20/NMS-P945 against human cancers in vitro and in vivo. EV20/NMS-P945 inhibited cell proliferation in multiple tumor cell lines in a target- and dose-dependent manner. In vivo EV20/NMS-P945 treatment effectively inhibited the growth of cancer cell-derived xenograft tumors in athymic nude mice. This novel ADC revealed a favorable pharmacokinetics, with terminal half-life (t_1/2_) and distribution volumes comparable to those of the naked antibody. Finally, administration to up to 8 mg/kg was well tolerated in cynomolgus monkeys. These data suggest that EV20/NMS-P945 may be a promising therapeutic agent for the treatment of HER-3 expressing tumors.

## 2. Results

### 2.1. Generation and Characterization of EV20/NMS-P945

**Figure 1:**
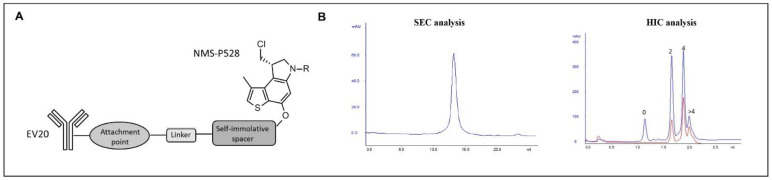
Generation and characterization of EV20/NMS-P945 anti-HER-3 antibody–drug conjugate (ADC). (**A**) Schematic representation of EV20/NMS-P945. (**B**) ADC characterization by size exclusion chromatography (SEC analysis, left panel), showing a high level of monomeric ADC obtained. ADC characterization by hydrophobic interaction chromatography (HIC analysis, right panel), showing different Drug-to–Antibody Ratio (DAR) species distribution. The peak at the lower retention time corresponds to the naked antibody (DAR 0), whereas the following peaks correspond to 2 and 4 drug loading (DAR 2 and DAR 4) with a lower amount of >4 DAR in the latest eluting peak. A low amount of the naked antibody was detected. HIC detection was performed at two different wavelengths, 220 nm (blue) for antibody detection and 320 nm (red) for bound drug detection.

NMS-P945, a payload comprising NMS-P528 (a DNA minor groove alkylating agent), a cathepsin-cleavable peptidic linker and a self-immolating spacer, was conjugated to EV20 to form EV20/NMS-P945 ADC (Figure 1A) through partially reduced interchain disulfide bridges. Complete characterization of the resulting product by hydrophobic interaction chromatography (HIC) and size-exclusion chromatography (SEC) showed that a monomeric ADC was obtained with a good conjugation level, reaching a DAR = 3.6 (Figure 1B). The EV20/NMS-P945 ADC was stable upon storage in PBS buffer up to 2 months at 4 °C, without aggregation or significant variation of conjugation levels, as evaluated by SEC and HIC (Supplemental Figure S1A,B). Plasma stability was also evaluated and the ADC, measured as total antibody by ELISA, remained stable at 37 °C up to 30 days in monkey and human plasma (Supplemental Figure S1C). A slightly faster decrease was observed when the ADC was determined as a conjugated antibody in monkey plasma by ELISA (Supplementary Figure S1C).

### 2.2. ADC Binding Properties

**Figure 2:**
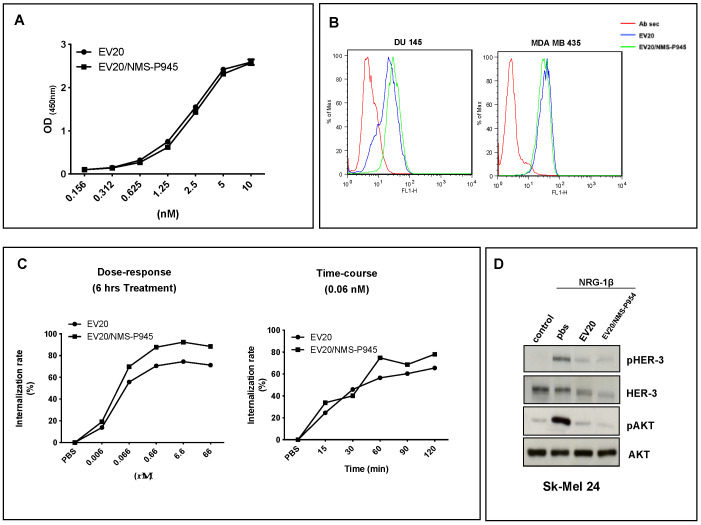
EV20/NMS-P945 shows similar properties in respect to the naked EV20 antibody. (**A**) In vitro binding affinity to the antigen of naked and NMS-P945-conjugated EV20 antibodies. ELISA was performed using as capture antigen human HER-3 extra cellular domain (ECD) and HRP-labelled goat anti-human IgG to detect bound EV20 or EV20/NMS-P945. (**B**) Cell binding affinity of naked and NMS-P945-conjugated EV20 was evaluated by flow cytometry using DU145 and MDA-MB-435 cells. Cells were harvested and stained with EV20 or EV20/NMS-P945 on ice, followed by staining on ice with PE-conjugate goat anti-human Fc as a secondary antibody. Ab sec: control cells stained only with PE-conjugate goat anti-human Fc. (**C**) Internalization ability of naked and NMS-P945-conjugated EV20 was evaluated by flow cytometry. A375M cells were exposed to increasing doses of EV20 or EV20/NMS-P945 for 6 h (left) or exposed for different times to 0.06 nM of EV20 or EV20/NMS-P945 (right), then cells were harvested and analyzed for surface HER-3 internalization. (**D**) Sk-mel 24 melanoma cells were serum-starved for 24 h, and incubated for 2 h in the presence or absence of 10 μg/mL of naked or conjugated EV20 mAb, before NRG-1β stimulation (10 min, 10 ng/mL). At the end of the incubation periods, cells were lysed and analyzed for pHER-3/HER-3 and pAKT/AKT protein levels by Western blotting.

As shown in Figure 2A,B, EV20/NMS-P945 was able to bind its target HER-3 with the same efficiency as that of the EV20 naked antibody, as evaluated by an in vitro binding assay (ELISA, Figure 2A) and by a cell binding assay (FACS (Fluorescence Activated Cell Sorting), Figure 2B). Moreover, the conjugation process did not alter the ability of the antibody to promote HER-3 internalization in a dose- and time-dependent manner, measured in terms of the percentage of receptor loss on the cell surface by FACS analysis (Figure 2C). Finally, the ability of the antibody to hamper receptor signaling was shown not to be altered by the conjugation process as evaluated in Sk-mel 24 cells stimulated with HER-3 ligand NRG-1β in the presence of NMS-P945-conjugated or naked EV20 (Figure 2D).

### 2.3. In Vitro Cytotoxic Activity of EV20/NMS-P945

**Figure 3:**
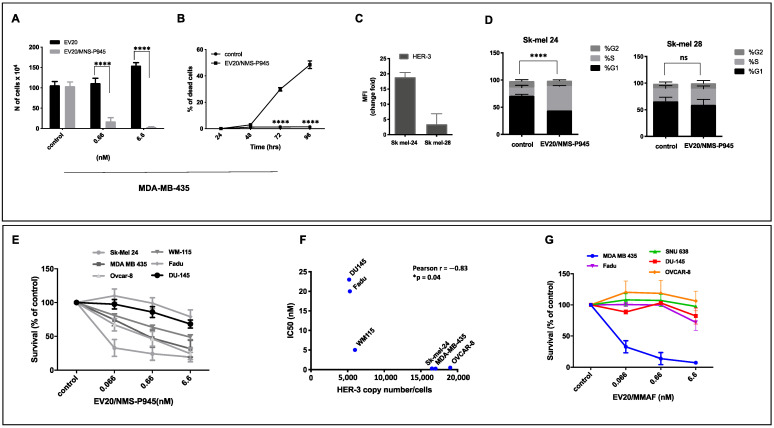
EV20/NMS-P945 possesses potent and target-dependent cell killing activity in vitro. (**A**) MDA-MB-435 melanoma cells were treated with the indicated doses of EV20 or EV20/NMS-P945 in complete medium. After five days, cells were harvested and several fields in the hemocytometer were counted. Mean +/− SD (*n* = 3). Significance was calculated by two-way ANOVA, followed by Bonferroni’s post hoc test (**** *p* < 0.0001). (**B**) Trypan blue staining of melanoma MDA-MB-435 cells treated for the indicated times with 0.6 nM of EV20/NMS-P945. Percentage of dead cells is presented. Mean +/− SD (*n* = 3). Significance was calculated by two-way ANOVA, followed by Bonferroni’s post hoc test. (**** *p* < 0.0001). (**C**) Sk-mel 24 and Sk-mel 28 cells were analyzed by flow cytometry for surface expression of HER-3 (left). Fold change of mean fluorescence intensity (MFI) is presented. (**D**) Cells were treated for 48 h with 0.6 nM of EV20/NMS-P945 and cell cycle was analyzed using propidium iodide DNA staining (right). Two-way ANOVA, followed by Sidak’s multiple comparison test was performed using GraphPad Prism 8.0 software (**** *p* < 0.00001 for G1% and S% differences). (**E**) Cell killing was evaluated with MTT assay on a panel of HER-3+ cancer cell lines treated with increasing doses of EV20/NMS-P945 for 120 h. Percentage of survival over control is represented. Mean +/− SD (*n* = 3). IC50 (Inhibit Cellular Proliferation by 50%) values were calculated using GraphPad Prism 5.0 software. *p*-values were calculated by two-way ANOVA analysis, followed by Bonferroni’s post hoc test and are reported in Table 1. (**F**) HER-3 expression in terms of receptor copy number and EV20/NMS-P945 IC50 correlation. Pearson coefficient was calculated using GraphPad Prism 8.0 software (Pearson r: −0.83; * *p* = 0.04). (**G**) Cell killing was evaluated with MTT assay on a panel of HER-3+ cancer cell lines treated with increasing doses of EV20/MMAF for 120 h. Percentage of survival over control is represented. Mean ± SD (*n* = 3). *p*-values were calculated by two-way ANOVA, followed by Bonferroni’s post hoc test and are reported in Table 1 (Supplementary Materials).

Next, we analyzed the potency and the target-dependence of EV20/NMS-P945’s cell-killing activity in vitro. Initial testing was done on HER-3-positive MDA-MB-435 melanoma cells. A five-day exposure of cells to EV20/NMS-P945 resulted in significant, dose-dependent cell growth inhibition, evaluated by cell counting. The naked antibody had no effect in the same assay conditions (Figure 3A). A time-course of EV20/NMS-P945 treatment at the sub-nanomolar concentration (0.6 nM) on MDA MB 435 cells produced a remarkable increase in cell death, starting from 72 to 96 h of treatment, as demonstrated by Trypan blue staining (Figure 3B). Then, we compared two melanoma cell lines; Sk-mel 24 and Sk-mel 28 (Figure 3C), for HER-3 expression on the plasma membrane. HER-3 expression resulted in much higher in Sk-mel 24 compared to Sk-mel 28 and the level of HER-3 expression was correlated to cell line sensitivity to the ADC. Indeed, we observed that a low concentration (0.6 nM) of the ADC induced a significant cell cycle block in the S phase in highly HER-3-expressing melanoma cells (Sk-mel 24), but not in low HER-3-expressing melanoma cells (Sk-mel 28) (Figure 3D).

Further testing of the ADC cytotoxic activity on a panel of tumor cell lines with various levels of HER-3 expression by the MTT assay revealed a significant positive correlation between surface HER-3 receptor density and cell killing activity (Figure 3E,F). Of note, although the cytotoxic activity of EV20/NMS-P945 was similar to that of EV20/MMAF in MDA-MB 435 melanoma cells, a superior activity of EV20/NMS-P945 was observed in cells originating from stomach (SNU638), prostate (DU145), ovary (OVCAR 8) and head and neck cancers (Fadu), indicating that the novel ADC possesses a broader antitumor activity (Figure 3G).

Overall, these results indicate that EV20/NMS-P945 is endowed with a potent and target-dependent cell-killing activity.

### 2.4. In Vivo Efficacy of EV20/NMS-P945

**Figure 4:**
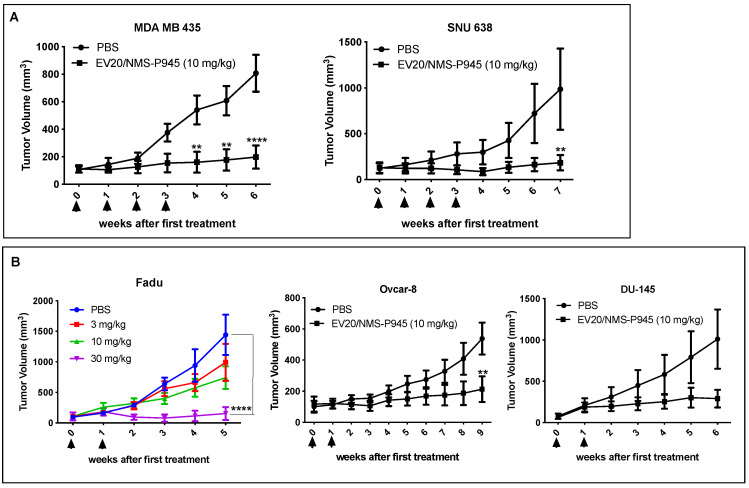
EV20/NMS-P945 displays therapeutic efficacy in a broad range of tumor types. (**A**) MDA MB 435 (2 × 10^6^ cells;) and SNU638 (3 × 10^6^ cells) cells were injected into the right flank of recipient mice; once established tumors had reached the approximate volume of 100 mm^3^, animals were divided into size-homogeneous groups (MDA MB 435: PBS (*n* = 6); EV20/NMS-P945 (*n* = 5). SNU638: PBS (*n* = 5); EV20/NMS-P945 (*n* = 5)) and received four intravenous injections, one per week, of EV20/NMS-P945 at the dosage of 10 mg/kg. Vehicle alone (PBS) was injected into the control mice. Mean tumor volume +/− SEM is presented. Arrows indicate treatment administration. (**B**) Fadu (2 × 10^6^ cells), OVCAR8 (2 × 10^6^ cells) and DU-145 (2 × 10^6^ cells) cancer cells were injected into the right flank of recipient mice; once established tumors had reached the approximate volume of 100 mm^3^, animals were divided into size-homogeneous groups (Fadu: PBS (*n* = 5); EV20/NMS-P945 3 mg/kg (*n* = 5); EV20/NMS-P945 10 mg/kg (*n* = 5); EV20/NMS-P945 30 mg/kg (*n* = 5). OVCAR8: PBS (*n* = 7); EV20/NMS-P945 (*n* = 6); DU145: PBS (*n* = 9); EV20/NMS-P945 (*n* = 5)) and received two intravenous injections, one per week, of EV20/NMS-P945 at the indicated dosage for Fadu and 10 mg/kg for the other xenografts. Vehicle alone (PBS) was injected into the control mice. Mean tumor volume ± SEM is presented. Arrows indicate treatment administration. *p*-values were calculated by two-way ANOVA, followed by Bonferroni’s post hoc test. (** *p* < 0.01; **** *p* < 0.0001).

EV20/NMS-P945 in vivo efficacy was evaluated in a panel of xenograft models expressing different amounts of HER-3. As preliminary approach, the ADC was used at a fixed dose of 10 mg/kg once weekly for a total of four injections. A potent tumor growth inhibition was observed in xenografts derived from melanoma cells (MDA-MB-435) and gastric cancer cells (SNU638; Figure 4A). Interestingly, even when reducing the schedule of ADC administrations from QWX4 to QWX2, a significant and dose-dependent tumor growth inhibition was observed in head and neck (H&N) (Fadu) and ovarian (OVCAR-8) xenograft models (Figure 4B). A trend toward a growth inhibition was observed in xenografts derived from prostate (DU-145) cancer cells (Figure 4B). EV20/NMS-P945 was well tolerated in mice, as no significant body weight loss was observed during treatment in all xenograft experiments (Supplemental Figure S2).

Moreover, the therapeutic activity of the ADC was evaluated in a model of xenografts derived from pancreatic cancer cells (BxPC-3) with a high tumor burden (about 500 mm^3^). A single injection of EV20/NMS-P945 at 40 mg/kg produced a significant reduction in tumor growth, with no signs of toxicity, as evaluated by body weight loss (Supplemental Figure S2) and hematological and biochemical analysis of blood samples obtained 48 h after ADC administration (Supplemental Figure S3).

In summary, EV20/NMS-P945 exerts a potent antitumor activity in a broad range of tumor types expressing different levels of HER-3, with no evidence of toxicity in mice.

### 2.5. Safety and Pharmacokinetic Profile of EV20/NMS-P945 in Cynomolgus Monkey

To gain insight into the potential use of this novel HER-3-targeting ADC for clinical purposes, we conducted an exploratory safety and pharmacokinetic study in the cynomolgus monkey.

**Figure 5:**
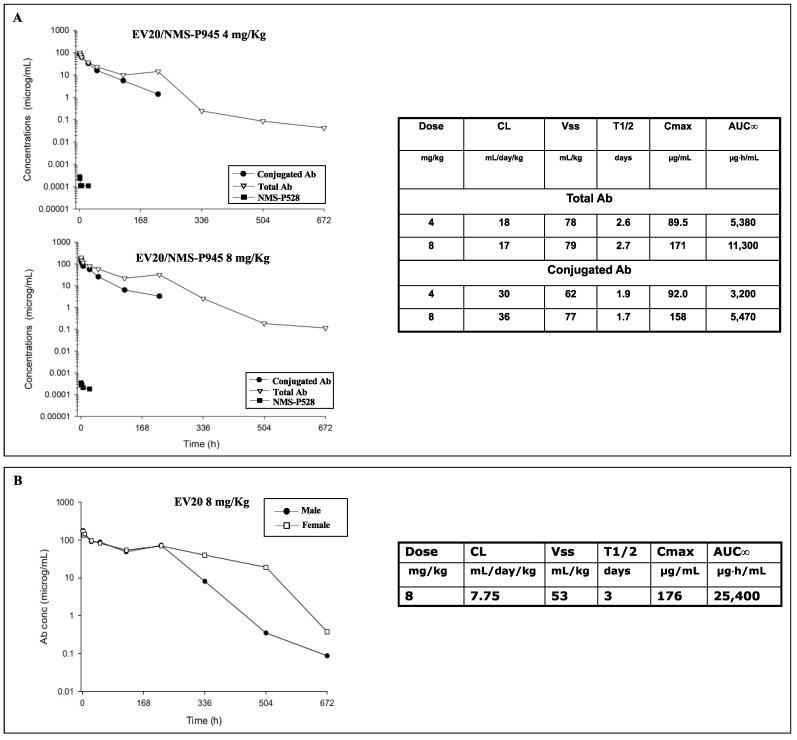
EV20/NMS-P945 pharmacokinetic study in cynomolgus monkeys. Two cynomolgus monkeys were injected with 4 and 8 mg/kg of EV20/NMS-P945 (**A**) or with 8 mg/kg of naked EV20 (**B**) by intravenous injection. Plasma samples were collected at the indicated time points post administration and non-competitive sandwich ELISA was performed. Total and conjugated antibodies were quantified using an anti-human IgG and an anti-NMS-P945 antibody as coating agents, respectively; naked antibody was quantified using an anti-human IgG antibody, whereas free NMS-P528 was determined by LC/MS-MS analysis. Plasma pharmacokinetic parameters of conjugated and total antibody after treatments with 4 and 8 mg/kg of ADC (**A**) and after treatment with 8 mg/kg of naked EV20 (**B**) are shown in the tables.

First, we performed a comparative tissue cross-reactivity (TCR) analysis using a panel of normal human and cynomolgus monkey tissues. As reported in Table 1, staining of the tissues with MP-RM-1 (murine analogue of the EV20) [20] revealed patterns of HER-3 expression that were superimposable between human and monkey tissues. For safety evaluation, the ADC was administered intravenously as a single injection at the doses of 4 and 8 mg/kg and the naked EV20 antibody was administered at a single dose of 8 mg/kg in two cynomolgus monkeys, one male and one female. No deaths and no abnormalities were recorded during clinical observations, as well as no body weight loss and no reduction of food intake. Hematology and serum chemistry were also evaluated. The observed changes were considered to be normal fluctuations for the species without any toxicological meaning. Indeed, serum chemistry variations observed were minimal in extent, not dose-related and mostly present in one sex (Supplemental Figure S4). Pharmacokinetic analysis revealed that the terminal half-life (t_1/2_) (2.6–2.7 days) and the volumes of distribution (78–79 mL/kg) of the total antibody were comparable or marginally lower than those of the naked antibody (t_1/2_ 3 days, Vss 53 mL/kg). The free toxic moiety NMS-P528, evaluated by LC/MS-MS analysis, was present in plasma at very low levels at an early time and it was no longer measurable 24 h after ADC dosing (Figure 5A,B).

Overall, the total and the conjugated antibody profiles are in line with the ones measured for other ADCs administered to monkeys. Kadcyla’s half-life is indeed between 4.6–5.2 days with a clearance of 9.4–10.5 mL/day/kg and a volume of distribution of 68–70 mL/kg when the ADC is administered to monkeys at 30 mg/kg, whereas Adcetris’s half-life after administration to monkeys at 3 mg/kg is between 1.6 and 2.7 days, with a clearance of 14.3–21.4 mL/day/kg [21].

## 3. Discussion

Over the past two decades, there has been a significant increase in interest in developing new cancer therapeutic agents specifically targeting and hampering the HER-3 receptor [6]. There are essentially two reasons behind this therapeutic strategy: first, HER-3 is overexpressed in many cancers [5]; second, the receptor acts as the main hub for escape mechanisms during the emergence of resistance to anticancer drugs [22,23,24]. In addition, somatic HER-3 oncogenic mutations have been identified in gastric and colorectal cancer patients [25]. Initial strategies adopted to block HER-3 activity included the use of small-molecule inhibitors (such as lapatinib, erlotinib, gefitinib, afatinib and neratinib), which are able to abrogate its dimerization partners’ kinase activity, or direct targeting of its extracellular domain through the blocking of monoclonal antibodies [26]. In particular, a panel of naked antibodies has been developed and entered into clinical trials both as a monotherapy and/or in combination with approved anticancer drugs known to induce HER-3 receptor upregulation [7]. The results of these studies showed no therapeutic improvement. More recently, the use of antibody–drug conjugates (ADCs) has been proposed as a different therapeutic approach to target HER-3-positive tumors efficiently. ADCs may be developed using monoclonal antibodies with high internalization capacity, as they mediate the efficient delivery of the conjugated, potent toxins inside target tumor cells [2].

To date, two HER-3-targeting ADCs have been developed. One of these is based on MMAE conjugated to the anti-HER-3 antibody 9F7–F11. This ADC, which is at the preclinical stage, was able to improve chemoradiation in PDAC [27]. A second ADC, named U3-1402, is composed of a humanized anti-HER-3 IgG1 antibody (patritumab) coupled to a novel membrane-permeable topoisomerase-I inhibitor DXd (DX-8951 derivative) through an enzymatically cleavable peptide-linker. U3-1402 is currently in Phase I/II clinical trials in metastatic breast, colorectal and non-small cell lung cancer [28,29,30].

Recently, we have developed HER-3-targeting ADCs by conjugating monomethyl-Auristatin F (MMAF) to humanized EV20 antibody using both a non-cleavable and (val-cit)-cleavable linker. These ADCs, called EV20/MMAF and EV20-sss-vc/MMAF respectively, displayed potent antitumor activity in melanoma and breast carcinoma resistant to anti-HER-2 therapies [13,14] and liver cancer [28]. Here, we aimed to increase the potential of EV20 as a scaffold for the specific delivery of cytotoxic agents by generating EV20/NMS-P945, which is composed of EV20 coupled to the novel thienoindole derivative NMS-P528 through a peptidic cleavable drug linker and a self-immolating spacer. A similar payload and conjugation chemistry has recently been successfully applied for the generation of an ADC targeting the ALK receptor in neuroblastoma [31] and HER-2 [17]. The results presented here demonstrate that EV20/NMS-P945 is obtained as a monomeric ADC with an average DAR of 3.6. The ADC has proven to be stable in storage buffer up to 2 months at 4 °C and in monkey and human plasma at 37 °C up to 30 days. Compared to the naked antibody, EV20/NMS-P945 ADC maintained the same in vitro and in vivo binding activity, internalization properties and ability to hamper receptor signaling.

As for ADC potency, EV20/NMS-P945 displayed a potent and target-dependent antitumor activity in vitro, which was positively correlated to surface HER-3 receptor density, in a wide range of solid human cancers. Importantly, this novel ADC showed in vitro superior cytotoxic activity compared to EV20/MMAF in some HER-3 positive solid tumors like gastric, ovarian, head and neck and prostatic cancer. A possible explanation could lie in the fact that the thienoindole derivative NMS-P528 is highly active in heterogenous tumors because of the bystander effect exerted by the toxin in cancer cells with low cell doubling time due to its mechanism of action, thus representing a good therapeutic option for solid tumors. TCR analysis using MP-RM-1, murine analogue of EV20, showed a restricted HER-3 expression pattern among normal human tissues, which was superimposable with non-human primate tissues, demonstrating cross-reactivity of EV20 with monkey HER-3. An exploratory safety study in cynomolgus monkeys demonstrated that the ADC is well tolerated at the dosage of 4 and 8 mg/kg and possesses a favorable pharmacokinetic profile, similar to that of commonly measured ADC analytes [21], such as Adcetris and Kadcyla. Of note, EV20/NMS-P945 demonstrated high stability in vivo in monkey plasma and free toxin was detected at very low levels in plasma and was present no longer than 24 h after ADC dosing. Duocarmycin-based ADCs represent a good therapeutic strategy, as demonstrated by (vic-)trastuzumab duocarmazine (SYD985), constituted by anti-HER2 antibody conjugated with a cleavable linker-duocarmycin analog, vc-secoDUBA, which recently entered phase III evaluation in metastatic breast cancer [3].

In summary, the data presented here position EV20/NMS-P945 as a promising therapeutic agent for the treatment of HER-3-expressing cancers.

## 4. Materials and Methods

### 4.1. Reagents

Antibodies used in the present study were as follows: phosphorylated ErbB-3 (Clone 21D3, Tyr1289, #4791), ErbB-3 (Clone D22C5, #12708), phosphorylated Akt (Ser473, Clone D9E, #4060), Akt (#9272), all from Cell Signaling Technology, Inc. Neuregulin-1β (NRG-1β, #5218SC) was purchased from Cell Signaling Technology, Inc. (Danvers, MA, USA). EV20 antibody was produced as previously described [13]. MP-RM-1 antibody was produced as previously described [20]. Recombinant human ECD HER-3 was from ACROBiosystems (Bethesda, MD, USA). (3-(4,5-Dimethylthiazole-2-yl)-2,5 diphenyltetrazolium bromide (MTT) was purchased from Sigma-Aldrich Corporation (St. Louis, MO, USA).

### 4.2. Cell Lines

Prostate cancer (DU145 (HTB-81)), head and neck squamous cell carcinoma (Fadu (HTB-43)), pancreatic cancer (BxPC3 (CRL-1687)), melanoma (A375M (CRL-3222), MDA-MB-435 (HTB-131), Sk-mel 24 (HTB-71), Sk-mel 28 (HTB-72), WM115 (CRL-1675)), cells were purchased from American Type Culture Collection (Rockville, MD, USA). Ovarian cancer (OVCAR-8) were from the National Cancer Institute/National Institutes of Health (NCI/NIH; Frederick, MD, USA). Gastric cancer cells (SNU638) were purchased from the Korean Cell Line Bank (KCLB). All cell lines were cultured less than 3 months after resuscitation. The cells were cultured according to manufacturers’ instructions, using EMEM (Eagle’s Minimum Essential Medium) from Thermo Fisher Scientific, Inc., (Waltham, MA, USA) for DU145, Fadu, Sk-mel 28, Sk-mel 24 and WM115 cells, DMEM (Dulbecco’s Modified Eagle’s Medium) from Thermo Fisher Scientific, Inc. for A375M, and RPMI-1640 medium (Thermo Fisher Scientific, Inc.) for MDA-MB-435, OVCAR-8 and SNU638 cells, supplemented with 10% heat-inactivated fetal bovine serum (FBS, Invitrogen, Carlsbad, CA, USA), l-glutamine, 100 units/mL penicillin and 100 μg/mL streptomycin (Sigma-Aldrich Corporation, St. Louis, MO, USA), and incubated at 37 °C in humidified air with 5% CO_2_.

### 4.3. Generation of EV20/NMS-P945

NMS-P528 was conjugated to the EV20 antibody through a partial interchain disulfide reduction, followed by derivatization with the drug with the aim of reaching an average DAR > 3.5. The resulting ADC was characterized by size exclusion chromatography (SEC) to check for aggregated material, by hydrophobic interaction chromatography (HIC) to evaluate the distribution of the different isoforms, and by reducing and nonreducing SDS-PAGE to assess purity. Liquid chromatography–mass spectrometry analysis of the reduced product allowed for DAR determination.

### 4.4. EV20/NMS-P945 Stability Evaluation

For stability evaluation in storage buffer, EV20/NMS-P945 was incubated in PBS at two different concentrations: 2 mg/mL and 4 mg/mL at 4 °C for 1 week, 2 weeks, 1 month and 2 months. Aggregation state, protein concentration and drug–antibody ratio (DAR) were analyzed using SEC and HIC analysis. For stability evaluation in plasma, EV20/NMS-P945 at the concentration of 225 μg/mL was incubated in monkey and human plasma at 37 °C for 0, 3 h, 24 h, 3 days, 5 days, 8 days, 15 days, 22 days and 30 days and aliquots were taken and analyzed by sandwich ELISA assay. The amount of total antibody and ADC were assayed by sandwich ELISA using total anti-human IgG and an anti-NMS-P945 antibody as coating agents, respectively. A 96-well plate was coated with anti-human IgG (Bethyl Lab. Cat n A80-319A) or anti-NMS-P945 antibody (custom made). After overnight coating at 4 °C, the plate was blocked with 100 μL of 2% BSA in PBS containing 0.05% Tween 20 (PBS-T) with agitation at room temperature for 1 h. Subsequently, the solution was removed and each ADC sample was added to each well, and the plate was incubated at room temperature for 2 h. After each well was washed three times with 100 μL of PBS-T, 100 μL of goat anti-Human IgG-h + l HRP Conjugated Antibody (Bethyl Lab. Cat n A80-319P) was added. After being incubated at room temperature for 1 h, the plate was washed three times with 100 μL of PBS-T and 100 μL of 3,3′,5,5′-tetramethylbenzidine (TMB) substrate was added. After color was developed for 10−30 min, acid solution was added to each well and then the absorbance at 450 nm was recorded using a plate reader.

### 4.5. Immunohistochemistry

Normal frozen tissues from cynomolgus monkeys were purchased as microarrays from US, Biomax, Inc. For the immunohistochemical analysis, frozen tissue sections underwent immunoperoxidase staining by incubation overnight at 4 °C (refrigerator) with the anti-HER-3 MP-RM-1 monoclonal antibody (mAb). After removal of the primary mAb and washing twice with cold PBS, sections were stained utilizing the Vectastain ABC kit (Vector Laboratories, Inc., Burlingame, CA, USA) according to the manufacturer’s instructions, using chromogens aminoethylcarbazole and 3,3′-diaminobenzidine (DAB), as appropriate. Nuclear staining was done using filtered Mayer’s hematoxylin (Whatman paper 541) for 5 min, followed by washing of the slides with filtered distilled water (Whatman paper 541). Finally, slides were mounted in aqueous mounting medium with no sealing of the coverslips.

The experiments included appropriate negative (isotype-matched primary irrelevant mAb) and positive (archival tissue section displaying HER-3 expression) controls. Immunohistochemical findings were recorded blindly by two investigators. Normal tissues were scored as “negative” when no cellular or interstitial component was stained on a wide range of primary antibody concentrations (10–50 µg/mL). Normal tissues were scored as “positive” when unequivocal HER-3 staining was observed.

### 4.6. ELISA

Recombinant HER-3 extracellular domain (ECD) (1 μg/mL) was pre-coated overnight at 4 °C on 96-well plates (NUNC Maxisorp modules). After blocking with 1% BSA in PBS for 1 h at room temperature, increasing concentrations (ranging between 0.15 nM and 10 nM) of naked EV20 or EV20/NMS-P945 were incubated for 1 h at room temperature. After several washes with PBS + 0.05% Tween-20, a goat anti-human IgG-HRP solution at a dilution of 1:5000 (A0170, Sigma-Aldrich Corporation) was added to each well and incubated for 1 h at room temperature. After washes, stabilized chromogen was added to each well for at least 10 min in the dark, then the reaction was stopped with the addition of H_2_SO_4_ 1N and the absorbance was read at 450 nm with an ELISA reader.

### 4.7. Internalization Assays

For flow cytometric analysis of internalization, A375M cells were plated in 12-well plates and grown in DMEM containing 10% FBS for 24 h. Cells were then incubated at 37 °C with increasing doses of EV20 or EV20/NMS-P945 for 6 h in a dose-dependent assay, and with 0.06 nM of EV20 or EV20/NMS-P945 for the indicated times in a time-dependent assay. In order to quantify surface HER-3 internalization, the cells were detached and stained with 10 μg/mL of EV20 as a primary antibody for 30 min on ice. After washing, cells were labeled with PE-conjugate goat anti-Human Fc at a dilution of 1:300 (H10104, Molecular Probes, Life Technologies, Carlsbad, CA, USA) for 30 min on ice. Analysis was performed using a FACSCantoII cytometer (BD Pharmingen, Franklin Lakes, NJ, USA). Data were analyzed with FlowJo software V10.7 (FlowJo, LLC, BD Pharmingen, Franklin Lakes, NJ, USA).

### 4.8. Flow Cytometry

For the cell binding assay, DU145 and MDA MB 435 cells were detached and labeled with 10 μg/mL of EV20 and EV20/NMS-P945 for 30 min on ice, followed by staining of 30 min on ice with PE-conjugate goat anti-Human Fc as a secondary antibody at a dilution of 1:300 (H10104, Molecular Probes; Life Technologies; Thermo Fisher Scientific, Inc.). For HER-3 surface expression analysis, Sk-mel 24 and Sk-mel 28 cells were harvested and labeled with 10 μg/mL of EV20 as a primary antibody for 30 min on ice. After washing, cells were labelled with PE-conjugate goat anti-Human Fc as a secondary antibody at a dilution of 1:300 (H10104, Molecular Probes; Life Technologies; Thermo Fisher Scientific, Inc.) for 30 min on ice. For evaluation of HER-3 copy number on the surface of a panel of cell lines, QIFIKIT (Quantitative Analysis Kit, Dako Agilent, Santa Clara, CA, USA) was used following the manufacturer’s instructions. Analysis was performed using a FACSCantoII cytometer (BD Biosciences).

### 4.9. Cell Cycle Analysis

After 48 h of treatment with 0.6 nM of EV20/NMS-P945, Sk-mel 24 and Sk-mel 28 cells were harvested and centrifuged for 5 min. Then, PBS was added to the cells, followed by 70% ethanol to bring the final volume to 0.5 mL. The cells were stored overnight at 4 °C. Cells were centrifuged and washed twice with PBS, before resuspending and staining them at 37 °C for 30 min with 50 µL of RNAse solution (10 mg/mL, R6513, Sigma-Aldrich Corporation) and then with 200 µL of propidium iodide solution (1 mg/mL, P4170, Sigma-Aldrich Corporation). Cell cycle analysis was performed using a FACSCantoII cytometer (Beckton Dickinson, Buccinasco, Italy). Data were analyzed using FlowJo software V10.7.

### 4.10. Cytotoxicity Assays

Cell proliferation was assessed by an MTT [3-(4,5-dimethyldiazol-2-yl)-2,5-diphenyl tetrazolium bromide] assay (Sigma-Aldrich), Trypan Blue staining (Sigma-Aldrich), and cell count. For the MTT assay, cell lines were seeded into 24-well plates at a density of 5 × 10^3^ cells/well in 500 µL of complete medium; they were treated with increasing doses of EV20/NMS-P945 or EV20/MMAF (ranging between 0.06 and 6.6 nM) and further incubated for 120 h. At the end of the incubation period, cells were incubated with 200 µL of MTT solution (medium serum-free with 0.5 mg/mL of MTT) for a further 2 h. After removal of the MTT solution, 200 µL of dimethyl sulfoxide (DMSO) was added to the wells for 10 min, and the absorption value at 570 nm was measured using a multi-plate reader. All experiments were performed in triplicate and the IC50 (Inhibit Cellular Proliferation by 50%) values were calculated using GraphPad Prism 8.0 software (GraphPad Software, Inc., San Diego, CA, USA).

For Trypan blue staining, after treatment with 0.6 nM of EV20/NMS-P945 for 24–48-72–96 h, MDA MB 435 cells were stained with 0.4% Trypan blue and were counted at various fields in the hemocytometer. The level of cytotoxicity was calculated as % of dead cells.

For cell count assay, MDA MB 435 cells were seeded in 6-well plates and exposed to naked EV20 and EV20/NMS-P945 at the doses of 0.6 and 6.6 nM for 120 h in a complete medium; then cells were trypsinized and counted using a hemocytometer. The average of cell counts from the triplicate was obtained, and the SD value was calculated statistically.

### 4.11. Western Blotting

Lysates from Sk-mel 24 cells exposed to the indicated treatments were prepared by washing cells twice in cold PBS followed by lysis with RIPA Buffer (50 mM Tris-HCl, 1% NP40, 0.1% SDS, 150 mM NaCl) supplemented with protease and phosphatase inhibitors (Sigma-Aldrich: Merck KGaA) for 10 min at 4 °C. Insoluble materials were removed by centrifugation (16,000× *g* for 10 min at 4 °C) and protein concentration was assessed using a Bradford assay. Equal amounts of protein (30 μg) were separated by SDS/PAGE on 10% polyacrylamide gel and transferred to nitrocellulose membrane. Membranes were blocked with 5% non-fat dry milk in PBS containing 0.1% Tween-20 for 1 h at room temperature and incubated with the indicated primary antibodies at the dilution of 1:1000 in PBS containing 0.1% Tween-20 overnight at 4 °C. After washing, membranes were hybridized for 1 h at room temperature with HRP-conjugated Goat anti-Rabbit IgG (STAR208P; Bio-rad Laboratories Inc., Berkeley, CA, USA) at a dilution of 1:20,000 in PBS containing 0.1% Tween-20. Detection was performed with a Plus-ECL chemiluminescence kit (Bio-Rad Laboratories, Inc.).

### 4.12. In Vivo Tumor Growth

Athymic CD-1 nu/nu female mice (5 or 7 weeks old) were purchased from Charles River Laboratories (Calco, Italy) and maintained at 22–24 °C and relative humidity, under pathogen-limiting conditions as required. Cages, bedding and food were autoclaved before use. Mice were provided with a standard diet and water ad libitum and acclimatized for 2 weeks before the start of the experiments. Housing and all procedures involving the mice were performed according to the protocol approved by the Institutional Animal Care and Use Committee of the Italian Ministry of Health (Authorization no. 292/2017-PR).

Xenografts were generated by subcutaneous injection into the right flank of mice of a range between 2 × 10^6^ and 5 × 10^6^ of cells (OVCAR-8, DU-145, SNU638, Fadu, MDA-MB-435 and BxPC-3) in 200 μL of PBS. When xenografts became palpable (approximately 100 mm^3^), animals were divided into groups to provide a similar range of tumor sizes for each group. The treated group received intravenous injections of EV20 or EV20/NMS-P945 in PBS at the indicated schedules and doses, whereas the control group received PBS only. The tumor volume was monitored weekly by a caliper and calculated using the following formula: tumor volume (mm^3^) = (length × width^2^)/2.

For the hematological and biochemical analysis, blood from mice treated with 40 mg/kg of EV20/NMS-P945 was collected after 48 h from administration. Samples were centrifuged at 2500× *g* for 10 min at 4 °C, and levels of alanine transaminase (ALT) and aspartate transaminase (AST) were quantified. Moreover, hematological analyses were performed including counts of red blood cells (RBC) and white blood cells (WBC), counts of platelets (PLT), levels of haemoglobin (HGB), haematocrit (HCT), mean corpuscular volume (MCV) and mean corpuscular haemoglobin (MCH).

### 4.13. Safety and Pharmacokinetic Study on Monkeys

EV20/NMS-P945 was administered to cynomolgus monkeys (one animal/sex/group) as a single IV dose at 4 or 8 mg/kg. One additional group of animals was administered with the naked antibody (EV20) at the dose of 8 mg/kg and one group with the vehicle with the same regimen. Animals were monitored for 4 weeks after administration; they were observed daily for mortality and clinical signs; qualitative food intake was recorded daily, and body weight was recorded weekly.

Hematology and clinical chemistry parameters were investigated pre-test and on days 3, 8, 15, 22 and 29 of the study; animals were sampled for 4 weeks for pharmacokinetics. The format of the ELISA was solid-phase and non-competitive, where the total antibody was extracted from the monkey matrix by means of a goat polyclonal anti-human IgG (Bethyl Lab. Cat n A80-319A) antibody bound to the surface of a 96-well high-binding microtiter plate. On the other hand, conjugated antibody was detected with an anti-NMS-P945 (custom made) antibody bound to the surface of a 96-well ELISA plate. After washing, blocking with 200 µL/well of TBS pH 8.0 containing 1% BSA, the bound analyte was tagged with a goat anti-Human IgG-h + l HRP Conjugated Antibody (Bethyl Lab. Cat n A80-319P). After an additional washing procedure, the HRP substrate TMB was added to allow the peroxidase reaction with the consequent substrate conversion to the colored product. The enzymatic reaction was stopped by the addition of 100 µL/well of Stop solution and finally the optical density (OD) at 450 nm was recorded. Conversion of the OD readings of samples into analyte concentrations was performed by interpolation with a calibration curve constructed by plotting the OD readings of calibration standards (CS), prepared by spiking known amounts of analyte into pooled blank monkey plasma, which was then diluted at the optimal MRD (Minimum Required Dilution). The most suitable mathematical model was used to fit the calibration standards results best and convert the OD reading values registered for the quality control (QC) samples into analyte concentrations. Finally, free NMS-P528 in monkey plasma was determined by LC/MS-MS analysis.

### 4.14. Statistical Analysis

For in vivo xenograft growth curves and cytotoxicity assays, *p*-values were calculated by two-way ANOVA, followed by Bonferroni’s post hoc test. (* *p* < 0.05; ** *p* < 0.01; *** *p* < 0.001; **** *p* < 0.0001). Statistical analysis of the cell cycle was performed using two-way ANOVA, followed by Sidak’s multiple comparison test. Experimental sample numbers (*n*) are indicated in the figure legends. All statistical analysis was performed with GraphPad Prism 8.0 software.

## Figures and Tables

**Table 1:** Frozen cynomolgus monkey multiple organ normal tissue array (US Biomax, Inc., FCY450) stained with MP-RM-1 antibody.

Immunohistochemical Staining		
Negative	Weakly Positive	Strongly Positive
Cerebrum	Parotid gland (ductal cells)	Pancreas (cell ducts)
Cerebellum	Colon	Kidney (proximal tubules)
Lung (pneumocytes)	Liver (hepatocytes)	
Skeletal and cardiac muscles	Glial cells	
Esophagus (basal cells)		
Stomach		
Small intestine		
Spleen		
Epidermis and adnexa		

## Data Availability

All data generated or analyzed during this study are included in this published article.

## Supplementary Materials

The following are available online at https://www.mdpi.com/article/10.3390/pharmaceutics13040483/s1, Figure S1: EV20/NMS-P945 stability in storage buffer was evaluated by SEC and HIC analysis performed on batches at 2 mg/mL and 4 mg/mL stored at 4 °C, to determine DAR and the percentage of free antibody in the preparation. Figure S2: Change in body weight in mice during the weeks after the start of treatments at the indicated doses in xenograft experiments. Arrows indicate treatment administration. Two-way ANOVA, followed by Bonferroni’s post hoc test, found results to be non-significant. Figure S3: (A) 3 × 10^6^ Bx-PC3 cells were injected into the right flank of recipient mice; once established tumors had reached the approximate volume of 400–500 mm3, animals were divided into two size homogeneous groups, and received one single injection of PBS or 40 mg/kg of EV20/NMS-P945. Tumor volume, assessed as described in the Materials and Methods section, is shown. Mean ± SEM is represented. Significance was calculated by two-way ANOVA, followed by Bonferroni’s post hoc test. (* *p* < 0.05; ** *p* < 0.01; **** *p* < 0.0001). (B,C) Hematological and biochemical analysis of blood samples obtained from control and treated mice 48 h after ADC injection. AST (aspartate aminotransferase); ALT (alanine aminotransferase); RBC (red blood cells); WBC (white blood cells); PLT (platelet); HGB (hemoglobin); HCT (hematocrit); MCV (mean corpuscular volume); MCH (mean corpuscular hemoglobin). Figure S4: Hematological (A) and biochemical (B) analysis of blood samples obtained from cynomolgus monkeys at indicated times after treatments. RBC (red blood cells); WBC (white blood cells); PLT (platelet); HCT (hematocrit); AST (aspartate aminotransferase); ALT (alanine aminotransferase); GGT (G-glutamyl transferase); ALP (alkaline phosphatase); TBIL (total bilirubin).
