# Supplementary material for: EV20/NMS-P945, a Novel Thienoindole Based Antibody-Drug Conjugate Targeting HER-3 for Solid Tumors
Source: Pharmaceutics. 2021 Apr 2;13(4):483. doi: 10.3390/pharmaceutics13040483 (PMC8066800; doi:10.3390/pharmaceutics13040483)
Supplement: Supplementary file 1 [file pharmaceutics-13-00483-s001.pdf]

pharmaceutics

MDPI

Supplementary Materials: EV20/NMS-P945, a Novel Thienoindole Based Antibody-Drug Conjugate Targeting HER-3 for Solid Tumors

Emily Capone, Rossano Lattanzio, Fabio Gasparri, Paolo Orsini, Cosmo Rossi, Valentina Iacobelli, Vincenzo De Laurenzi, Pier Giorgio Natali, Barbara Valsasina, Stefano Iacobelli and Gianluca Sala

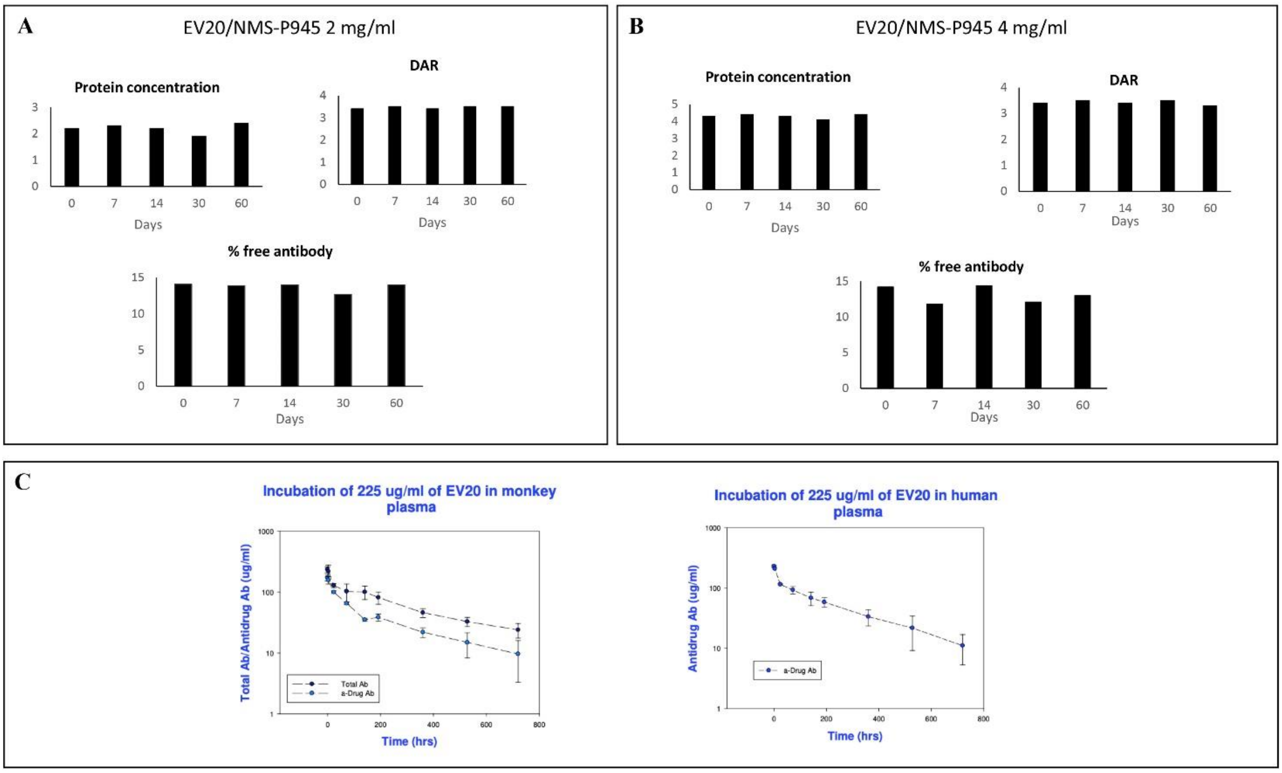

Figure S1. EV20/NMS-P945 stability in storage buffer was evaluated by SEC and HIC analysis performed on batches at 2 mg/mL and 4 mg/mL stored at 4 °C, to determine DAR and the percentage of free antibody in the preparation. Graphs indicating protein concentration, DAR and free antibody percentage over time at 2 mg/mL (**A**) and 4 mg/mL (**B**) are presented. (**C**) Plasma stability of generated ADC. After incubation of 225 µg/mL of EV20/NMS-P945 at 37 °C in monkey (left panel) and human plasma (right panel) for varying times, aliquots were taken and analyzed by means of the sandwich ELISA assay. For monkey plasma, total and conjugated antibodies were quantified using anti-human IgG and an anti-NMS-P945 antibodies as coating agents, respectively, whereas for human plasma, only the conjugated antibody was detected due to anti-idiotypic antibody unavailability.

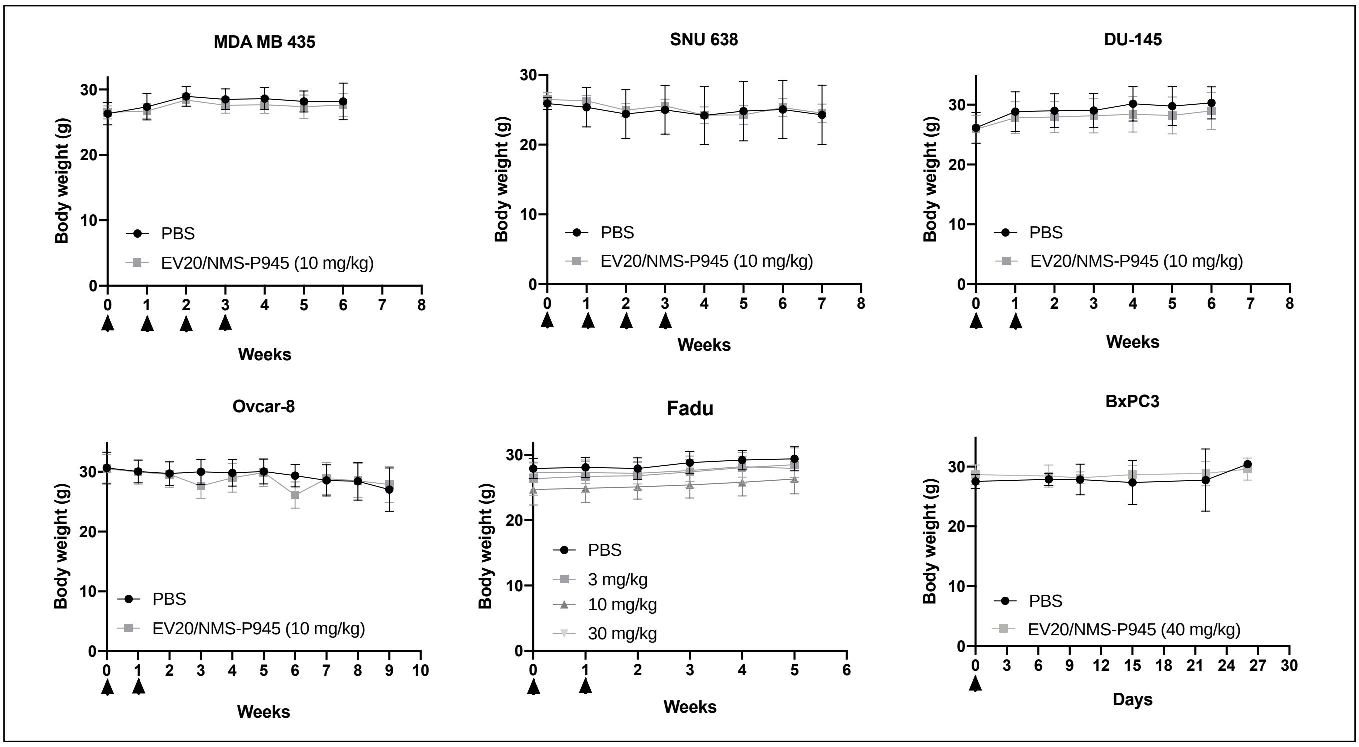

Figure S2. Change in body weight in mice during the weeks after the start of treatments at the indicated doses in xenograft experiments. Arrows indicate treatment administration. Two-way ANOVA, followed by Bonferroni's post hoc test, found results to be non-significant.

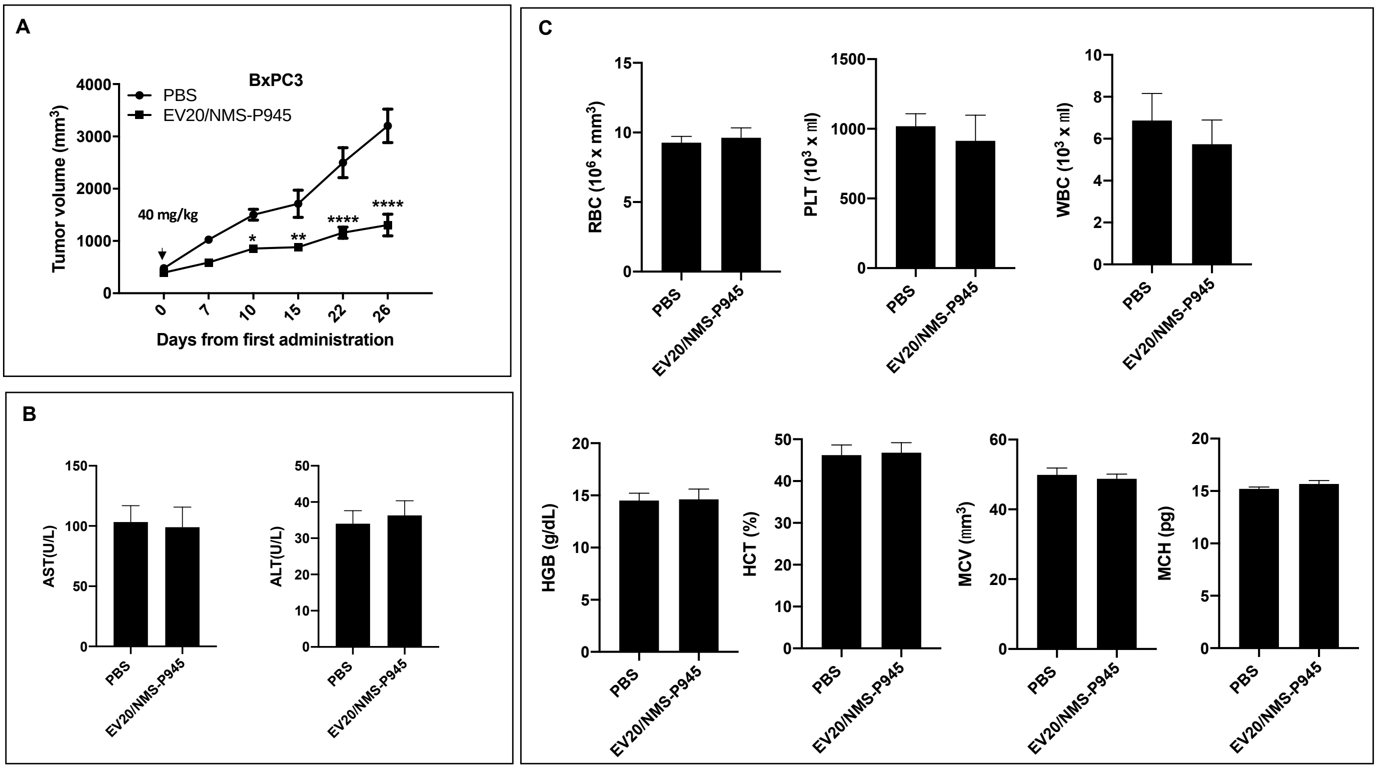

Figure S3. (**A**) 3 × 10[6] Bx-PC3 cells were injected into the right flank of recipient mice; once established tumors had reached the approximate volume of 400–500 mm^3, animals were divided into two size homogeneous groups, and received one single injection of PBS or 40 mg/kg of EV20/NMS-P945. Tumor volume, assessed as described in the Materials and Methods

section, is shown. Mean +/− SEM is represented. Significance was calculated by two-way ANOVA, followed by Bonferroni's post hoc test. (* $p < 0.05$; ** $p < 0.01$; **** $p < 0.0001$). (**B**,**C**) Hematological and biochemical analysis of blood samples obtained from control and treated mice 48 h after ADC injection. AST (aspartate aminotransferase); ALT (alanine aminotransferase); RBC (red blood cells); WBC (white blood cells); PLT (platelet); HGB (hemoglobin); HCT (hematocrit); MCV (mean corpuscular volume); MCH (mean corpuscular hemoglobin).

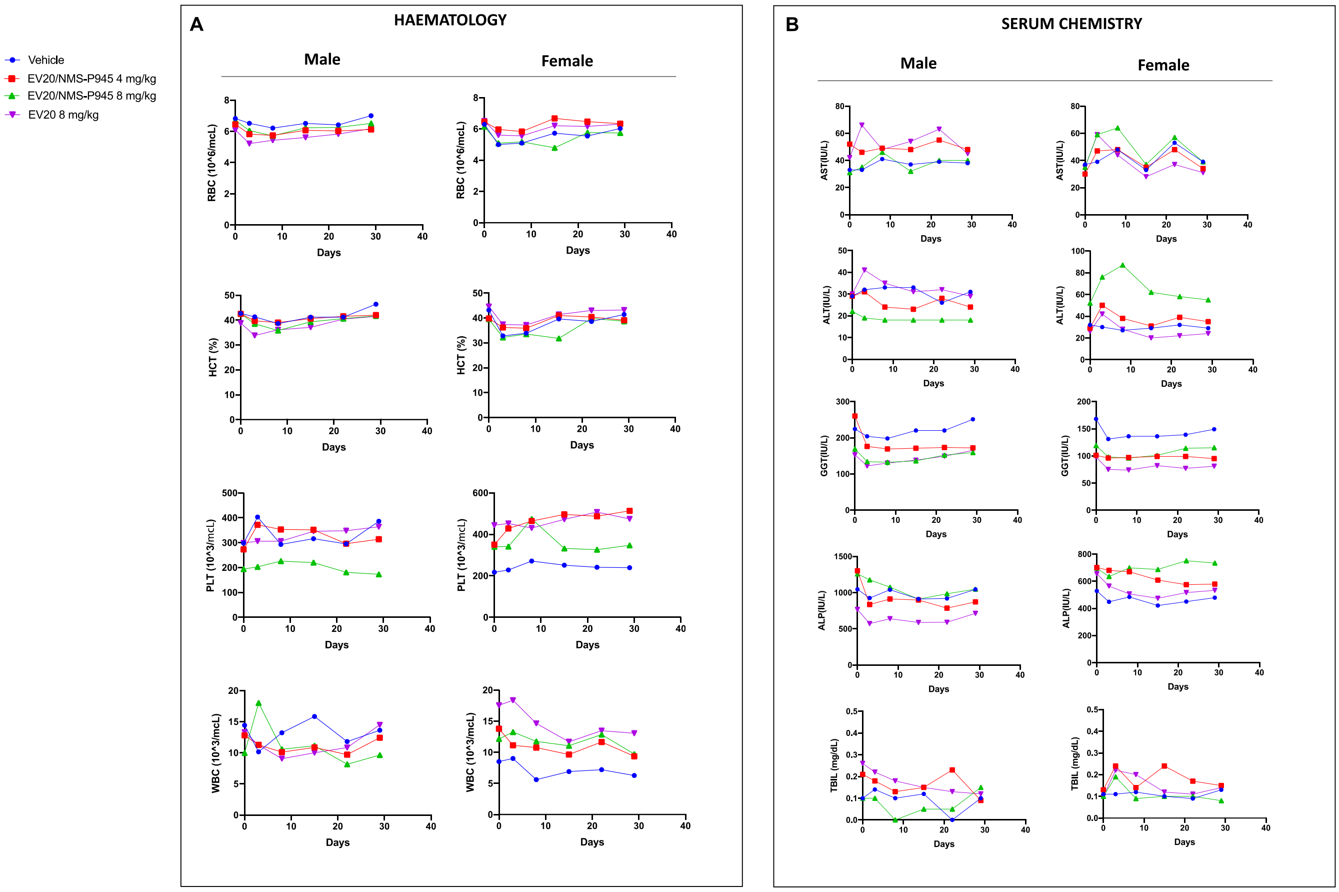

Figure S4. Hematological (**A**) and biochemical (**B**) analysis of blood samples obtained from cynomolgus monkeys at indicated times after treatments. RBC (red blood cells); WBC (white blood cells); PLT (platelet); HCT (hematocrit); AST (aspartate aminotransferase); ALT (alanine aminotransferase); GGT (G-glutamyl transferase); ALP (alkaline phosphatase); TBIL (total bilirubin).
